# The Ultraviolet Index Is Well Estimated by the Terrestrial Irradiance at 310 nm

**DOI:** 10.3390/s21165528

**Published:** 2021-08-17

**Authors:** Peter D. Kaplan, Emmanuel L. P. Dumont

**Affiliations:** 1 Shade, 111 Ideation Way, B511, Nutley, NJ 07110, USA; 2 Hackensack Meridian Center for Discovery and Innovation, 111 Ideation Way, Nutley, NJ 07110, USA

**Keywords:** ultraviolet index, ozone layer, atmospheric models, skin cancer risk, calibration, wearable sensor, photodiode

## Abstract

Ultraviolet (UV) exposure significantly contributes to non-melanoma skin cancer. In the context of health, UV exposure is the product of time and the UV Index (UVI), a weighted sum of the irradiance *I(λ)* over all wavelengths from *λ* = 250 to 400 nm. In our analysis of the United States Environmental Protection Agency’s UV-Net database of over 400,000 spectral irradiance measurements taken over several years, we found that the UVI is well estimated by 77 *I*_310_. To further understand this result, we applied an optical atmospheric model to generate terrestrial irradiance spectra and found that it applies across a wide range of conditions. An accurate UVI radiometer can be built from a photodiode covered by a bandpass filter centered at 310 nm.

## 1. Introduction

One of the most important risk factors for non-melanoma skin cancer is ultraviolet light (UV) exposure [1,2]. However, avoiding overexposure to UV remains difficult because UV does not correlate with visible brightness, its damage is wavelength dependent, and the harm is delayed by both hours (sunburns) and years (skin cancer). As a result, wearable UV monitors have the potential to help people assess their risk of UV overexposure in real-time. There are, however, currently no available sensors that are both sufficiently accurate and inexpensive for individual use [3,4]. In more recent reviews of wearable UV monitors, accuracy was not discussed [5,6]. To be accurate, UV sensors must replicate the wavelength-dependent skin sensitivity to UV, also called the “erythema action spectrum” [7]. When the erythema action spectrum is multiplied by the ambient ultraviolet spectrum, one obtains the UV Index (UVI), a unit adopted by the World Health Organization [8,9]. Here, we present a novel way to estimate the UVI. It was developed by analyzing over 400,000 UV spectra collected over 10 years at multiple sites [10]. We find that a narrow band irradiance detector at 310 nm is sufficient to measure the UVI [11]. Using a simple atmospheric model [12] to explore the range over which the result is applicable, we find that the strategy is likely to apply across essentially all terrestrial atmospheric conditions. This feasible, accurate, and wearable approach to UV-monitor design has promise for use in data-driven UV exposure management.

**Figure 1:**
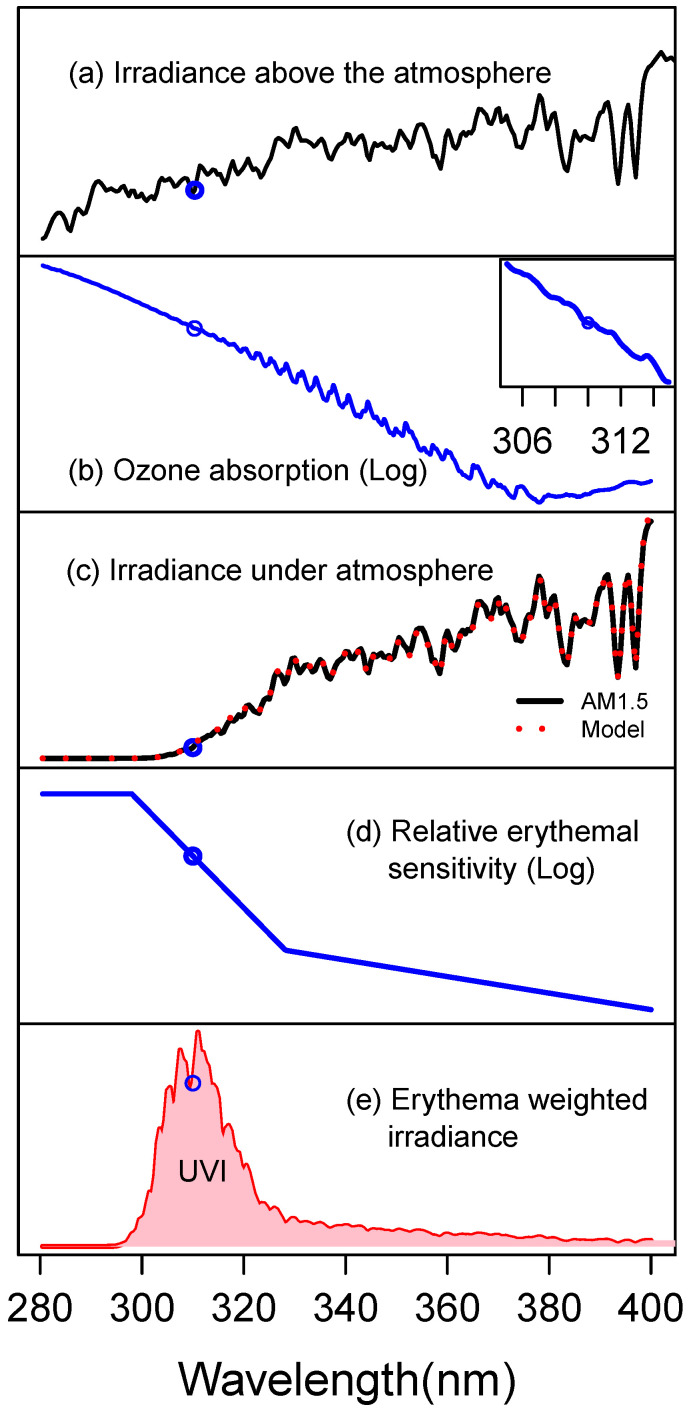
The solar spectrum and the wavelength dependence of its modification by the environment and contribution to the UV Index. (**a**) The AM0 solar spectrum (mW/m^2^/nm) is attenuated in the atmosphere by both aerosol loss (light scattering, not pictured) from water droplets including clouds and by absorption, mostly due to (**b**) ozone (cross-section in cm^2^/molecule). (**c**) The resulting AM1.5 solar irradiance on the earth’s surface, black line, and a fit mentioned in the discussion, red-dashed line, that quantitatively follows each wiggle in the solar spectrum (**d**) must be weighted by multiplying by the erythema action spectrum (dimensionless, equal to 1 at 280 nm), which is the relative sensitivity of skin to each wavelength. (**e**) The product is the biologically relevant erythema-weighted irradiance spectrum (erythemal mW/m^2^/nm). The UVI is the shaded area under this curve divided by 25. The value of each quantity at 310 nm is highlighted (blue dots) (**a**) and the black curve in (**c**) is drawn from ASTM–G159–98, [22]. The erythema action spectrum is ISO/CIE 17166:2019.

The UVI is calculated (Figure 1) by adding the erythema-weighted sun irradiance at each wavelength and dividing the total by a reference value of 25 mW/m^2^ [13]. By design, the UV Index is dependent on the local solar spectrum, which in turn is determined by instantaneous cloud cover and other environmental details. It is common to publish the UVI by day and by geographical region, for example, by zip code [14]. However, this approach fails to account for local and transient changes. Additionally, because skin damage is proportional to both the UVI and the duration of exposure, communicating the UVI without tracking actual exposure time may be misleading [8,15]. There have been many attempts to develop commercial, hand-held, or wearable low-cost UV dosimeters [6]. These detectors, however, have been largely inaccurate [4] as there is a significant mismatch between their spectral sensitivity and the erythema action spectrum (Figure 1d). Indeed, because there is much spectral variation by the latitude, altitude, time of day, season, local weather, and hyper-local environment, it is generally understood that any detector whose spectral sensitivity is not exactly the erythema action spectrum will not be able to accurately estimate the UVI [16]. In this report, we present a novel sensor design to reliably determine the UVI that is widely applicable across geographies, seasons, and atmospheric conditions. We find that an accurate detector can be designed using only irradiance near 310 nm. The result emerges from an analysis of a large database of spectral measurements of sunlight in several US locations across many years and is further validated by an atmospheric model.

The value of the UVI depends on four wavelength-dependent quantities: the solar spectrum above the atmosphere, the loss during transit due to aerosol scattering and the loss during transit due to ozone absorption, and, finally, the erythema action spectrum (Figure 1). The spectrum of solar light impinging on the atmosphere is relatively stable and well documented [17]. Loss of UV in the atmosphere is mostly due to ozone absorption and aerosol scattering primarily from water droplets, including clouds. The erythema action spectrum weights each wavelength according to its potential to damage skin [7,18].

Technical designs for UVI detectors have taken two routes. The first, pioneered by Robertson and Berger in 1976, uses a meter that responds to each wavelength according to the erythema action spectrum [19]. This approach is both expensive and bulky as it requires careful combinations of filters and detectors. The second, used by most consumer-grade detectors, is to use a UV-sensitive photodiode with the hope that, although spectrally mismatched, it can be accurate enough. The nature of UV spectral fluctuations, which we will discuss in detail below, has defeated this approach. To look for a new approach, we analyzed the US Environmental Protection Agency’s UV-Net database [20]. This database includes 400,000 UV irradiance spectra collected by atmospheric scientists across nine sites between 1996 and 2004, using Brewer spectrometers that collect data in 0.5 nm bandwidth channels from 286 to 363 nm. Sites range from near sea level to mountainous elevations and from urban to rural. UV-Net data have been integral to both atmospheric and clinical research [20,21].

## 2. Materials and Methods

The definition of UVI is

(1) $$\mathrm{UVI}=\frac{1}{25}\int^{\;}w\left(\lambda \right)I\left(\lambda \right)d\lambda ,$$

where *w*(*λ*) is the erythema action spectrum (EAS, Figure 1d) and *I*(*λ*) the local spectral irradiance in mW/m^2^/nm. To calculate the UVI numerically from the UV-Net solar spectra, we applied Equation (1) to each solar spectrum. The UVI is defined using the global (direct + diffuse) spectral irradiance.The output of a given sensor with a wavelength-dependent sensitivity *s*(*λ*) can be modeled as *M* = ∫ s(*λ*)I(*λ*)d*λ*. In use, detector current is fed into a calibration function *C*(*M*) to estimate *U_m_*, the UVI. In this paper, for simplicity, we use linear calibration models of *U_m_* = *C*_0_ + *C*_1_ *M*.

The accuracy of a detector is determined by calculating, in turn, its output *M*, the calibration function *C*(*M*) from a linear regression on *U*~*C*(*M*), and then using the coefficients of the regression to calculate the percent error for each measurement ϵ=100[(C(M)/U)−1]. The accuracy, *A*, as a function of the tolerance, *t*, is A(t)=ECDF(ϵ), the empirical cumulative distribution function, that is, the percentage of spectra with error less than a tolerance *t* as a function of that tolerance.

In the discussion, we develop an atmospheric model which includes the ozone content of the atmosphere in Dobson Units. One Dobson Unit corresponds to an ozone column height of 0.001 cm at standard temperature (272 °K) and pressure (1 atm):

(2) $$1DU=1000\frac{\mathrm{DU}}{\mathrm{cm}}\;1000\frac{{\mathrm{cm}}^{3}}{\mathrm{liter}}\;22.4\;\frac{\mathrm{liter}}{\mathrm{mole}-\mathrm{atm}}\;\frac{1}{N_{A}}\;\frac{\mathrm{mole}}{\mathrm{molec}}\;1\;\mathrm{atm}=3.721\cdot 10^{-17}z,$$

where *N_A_* is Avagadro’s Number and *z* is the concentration of ozone that appears in Equation (2). Fits to the model were performed using nls.lm() in the package “stats” of the R project for Statistical Computation.

## 3. Results

**Figure 2:**
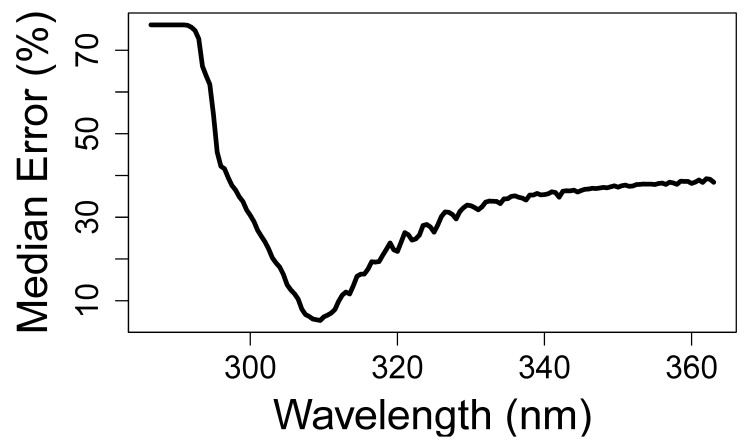
The median percent error in UVI prediction across all 405,000 UV–Net spectra, modeled as an ideally calibrated linear single-wavelength detector as a function of the detector wavelength. There is a sharp and narrow band of low error at 310 nm.

To investigate the role of each wavelength to UVI, for each spectrum, we calculated the UVI and, using the irradiance *I* at each wavelength *λ*, we performed a linear regression of *U* ~ *I_λ_*. To compare wavelengths, we used ϵ, the relative difference between the spectrum’s UVI and the UVI predicted by the linear fits. This metric was introduced by Correa et al. in the first study comparing the performance of hand-held UV sensors [4]. As a measure of accuracy, we calculated the percentage of measurements that the linear regressions predicted to within a given tolerance (%) of the ground-truth value. The variation of accuracy with wavelength is plotted in Figure 2. The model most commonly yielding both the lowest relative error and the highest overall accuracy is *U* = $76.6I_{310}-0.02$, 95% confidence intervals (76.62, 76.66) in units of inverse Irradiance or nm-m^2^/W and (−0.0166, −0.0145) UVI; R^2^ = 0.99. This result is surprising because it survives across all spectra in the database which include those with different ratios of UV-A to UV-B, across ozone fluctuations, and with all observed cloud and aerosol conditions. We know of no physical or optical argument that would lead to the conclusion that such a narrow-band indicator wavelength should exist.

**Figure 3:**
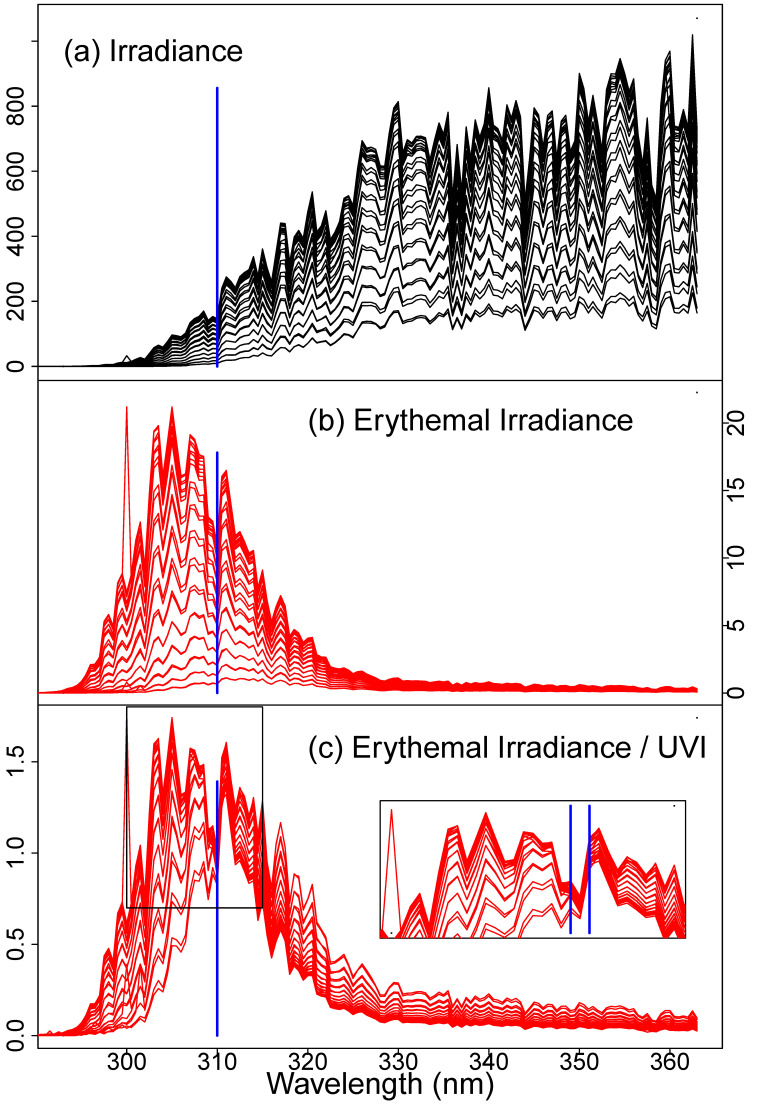
Graphical derivation of the result. (**a**) All UV spectra (mW/m^2^/nm) from Acadia National Park in Maine, US, on 21 June 2000, with UVI > 0.5. (**b**) The same spectra weighted by the erythema action spectrum (Figure 1d). (**c**) These spectra are normalized by UVI (mW/m^2^/nm/UVI). After normalization, the spectra collapse, nearly to a point, at 310 nm. The available code may be used by the reader to reproduce this figure for any location and date for which UV Net data were collected.

To illustrate this observation, we present a simple graphical derivation in Figure 3. We first plot (Figure 3a) all spectra from a single a UV-Net site over a single day for which the UVI exceeded 0.5. The spectra are complex, even after erythemal weighting (Figure 3b). This complexity is the underlying reason why there are so many discussions of the need to consider both UV–A and UV–B when considering skin risk and protection. However, when each weighted irradiance spectrum is divided by the UVI (Figure 3c), they converge at 310 nm, and only at 310 nm. The code in the supplementary material enables the reader to re-render Figure 3 using data from any day at any of the UV–Net sites. The importance of considering actual solar spectra is highlighted by the fact that these spectra vary through the day and do not, in any simple way, resemble any spectrum with fixed ratios of intensities at various wavelengths. Compare the spectra in Figure 3, for example, to the standard AM1.5 solar spectra (Figure 1c).

**Figure 4:**
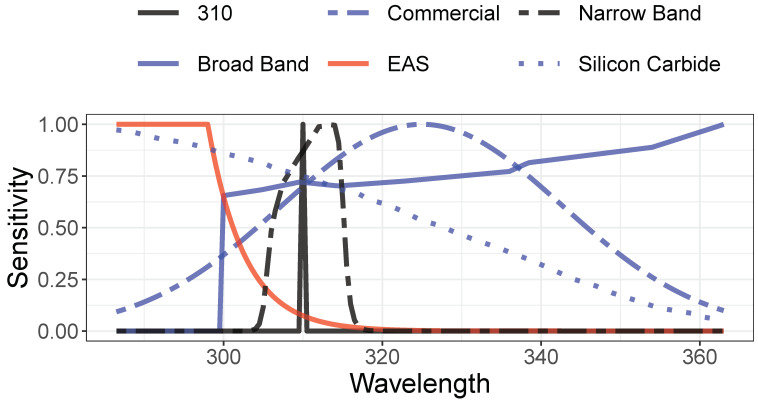
Spectral sensitivity curves of the filters modeled in Table 1. The theoretical ideal is the erythema action spectrum (red), 310 nm designs (black) include a narrow band and a more practical narrow band pass. Other UV-sensitive designs (blue) include broad band detection, silicon carbide, and a detector commercially marketed for estimating UVI.

To put this performance in the context of the accuracy of other detector designs, we modeled the performance of several designs. Their spectral sensitivity functions are displayed in Figure 4. The filters include 310 ± 0.5 nm, a more practical narrow band (NB) design consisting of a spectrally flat detector beneath a commercially available 310 nm bandpass (Alluxa 7365), a bare silicon carbide photodiode (SGS01S–18, SGLux, Berlin, Germany), a commercially packaged detector sold for measuring UVI (VEML6075, Vishay, Malvern, PA, USA), and a broad band (BB) UV detector which we model as a combination of a flat bandpass filter from 300 to 400 nm over a commercial detector (OPT3002, Texas Instruments, Dallas, TX, USA). The wavelength-dependent sensitivity for each commercial device is extracted from its publicly available data. For each detector *d* and each UV–Net spectrum *i*, we calculated *M_di_*, the current expected under each of the UV–Net spectra, built a linear model *U*~*M_d_*, and tabulated the expected median detector current, error, and accuracy at 10% tolerance, as described in Methods above. The results, in Table 1, include the comparative signal strength, mean error, and percentage of measurements accurate to within 10% for each detector.

## 4. Discussion

Any practical implementation of the 310 nm detector will require considerations of engineering realities. Detector output current, the product of detector sensitivity, filter transmission, and spectral irradiance, is a critical design factor. To explore the reduction in performance for using a real-world detector based on commercially available bandpass filters, we modeled the signal levels and accuracy and expected that implementations designed using the 310 nm insight would perform well. Notably, the detector current achievable with a narrow band filter is 30% of the current expected from a perfect EAS filter due to the exponential cut-off in sensitivity for wavelengths > 298 nm. A complete commercial design is beyond the scope of this paper.

To understand the limitations and applicability of a 310 nm detector, as well as to illustrate the reasons that such a filter might work, we use a simple model for surface UV irradiance. The model, based on a simple single-layer atmosphere, was published by Huber and collaborators [12]. In the Huber Model, the surface irradiance $I_{m}$ depends on only three adjustable parameters, *A*, *B*, and *z*:

(3) $$I_{m}\left(\lambda \right)=exp\left[-A+B\left(\lambda -320\right)-z\frac{\sigma \left(\lambda \right)}{cos\left(\phi \right)}\right]I_{solar}\left(\lambda \right)$$

**Figure 5:**
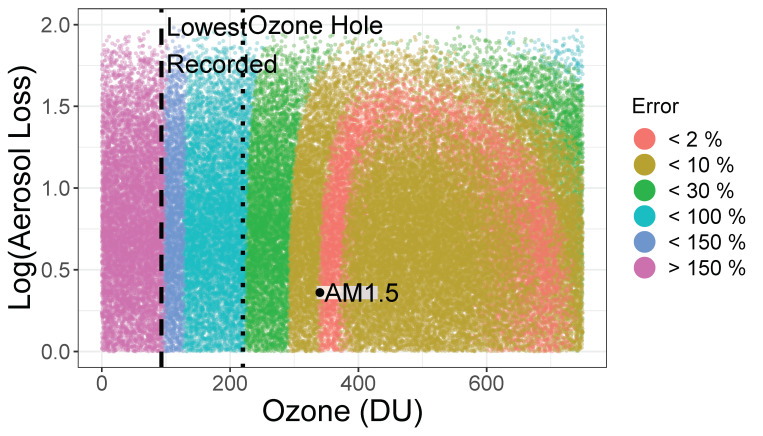
The error predicted by an atmospheric optical model (Equation (2)) for using the approximation UVI = 76.6 *I*_310_ nm. We indicate the location of the standard conditions, AM1.5 Spectrum (Figure 1c) in this parameter space (black dot).

*A* (>0) is the aerosol loss, *B* (in nm^−1^) grossly captures the wavelength dependence of Rayleigh scattering from water droplet size of various sizes, and z (>0, in molecules/cm^2^) is the amount of atmospheric ozone. The model in Equation (2) uses several known quantities including σ, the ozone cross-section (Figure 1b), an adjustment to the optical path through the ozone layer 1/cos(ϕ) where ϕ (between 0 and π/2) is the solar zenith angle, and $I_{solar}$, the air mass zero (AM0) solar irradiance spectrum above the atmosphere (Figure 1a). The model has a remarkable ability to capture the detailed spectral variation of solar UV light. In Figure 1c, we show the overlap of this model’s predictions on a reference irradiance spectrum. We fit each of the 405,000 UV-Net spectra to Equation (2). The resulting distribution was used to estimate the naturally occurring range of parameters *A*, *B*, and *z*. We then generated a sample of points in the three-dimensional parameter space *A*, *B*, and *z* covering the range centered on the observed values and extending along each axis by two standard deviations. For each set of parameters (*A_j_*, *B_j_*, *z_j_*) we calculated the full spectrum, calculated the UVI, and extracted the intensity at 310 nm *I_j_*. In Figure 5, we show the error in the *A*–*z* projection of this parameter space by coloring each point according to its engineering accuracy, $\left|\left[77I_{j}/U\right]-1\right|$. Similar projections in the (*A*, *B*), and (*B*, *z*) planes show no additional clear boundaries for the model. This diagram can form the basis of an expected range of accuracy for a 310 nm design. Naturally occurring ozone levels are generally in the range of 250–500 Dobson Units (DU) and average 300, the equivalent of a 3 mm thick layer of ozone gas at zero degrees celsius and 1 atmosphere. For reference, at ozone levels below 220 DU (ozone holes), the model begins to underestimate the UVI. The lowest observed ozone level is 93 DU. We expect that using solar irradiance at 310 nm to estimate the UVI should be a valid approach over a wide range of clear and cloudy skies. The Huber Model predicts that the conditions under which the relationship *U* = $77I_{310}$ is off by more than a factor of 1.5 rarely occur.

## 5. Conclusions

We propose a design for a simple linear detector for the measurement of the UV Index. The detector design is only the first step in building a useful sensor. Limitations of this paper include limiting our analysis to linear models and single-sensor measurement approaches. Geometric factors, including the need to orient the detector and the correct angular sampling of input light, have not been considered. In practice, real-world detector sensitivities may slightly degrade performance (Figure 4). On the other hand, it is likely that the simple linear estimate based on a single diode can be improved by introducing more complex functional forms and supplementary detectors. From the point of view of sensor engineering, however, starting with the most accurate achievable linear detector is desirable.

The goal of UVI measurement including wearable UVI measurement is to support improved healthy UV behavior. The UVI is a risk communication tool. Most explanations of UV risk are complex as they include the discussion of risk from UV-A and UV-B as well as the duration of exposure. This complex messaging is required, in part, because of the need to highlight that UV-A presents continuing risk even when UV-B is attenuated, for example when the sun is low in the sky and under cloud cover. This work highlights the fact that natural variations in UV-A and UV-B are captured by the UV Index. It remains proportional to *I*_310_ even as the ratio of UV-A to UV-B varies through the day (Figure 3). It may be that simpler and more meaningful risk communication could be made with just one number, the personal UVI. 

Reducing any spectral analysis to one or a few wavelengths is a considerable practical simplification. For example, low-cost blood oxygen measurements depend on the ability to operate with two wavelengths. Here, we have described a simple relationship between the irradiance of the sun at 310 nm and the response of human skin to UV. We have also described a useful method to evaluate the design performance of UV sensors using their spectral sensitivity and a large database of publicly available spectra. We confirmed that our result is sensible in that it is consistent with the existing understanding of UV irradiance using a simple atmospheric model applied to a wide range of conditions. The use of this model provides additional confidence that a detector designed around a detector with peak sensitivity at 310 nm will produce valid results over weather conditions and geographies not surveyed by UV Net.

## 6. Patents

Dumont, E.L.P.; Kaplan, P.D. Methods, systems, and apparatuses for accurate measurement of health-relevant UV exposure from sunlight. U.S. Patent No. 10,876,886.

## Figures and Tables

**Table 1:** Modeled performance of spectral sensitivity functions in Figure 4. Median signal is relative to EAS. Accuracy is the percentage of spectra whose UVI is predicted with an error less than 10%.

Detector	Median Signal	Median Error (%)	Accuracy at 10% Tolerance
EAS	1	0	100
310 ± 0.5 nm	0.3	6	66
Narrow Band	7	8	60
Broad Band	200	33	19
Silicon Carbide	74	27	23
Commercial Example	140	30	21

## Data Availability

The United States Environmental Protection Agency (EPA) UV-Net was a network of Brewer spectrometers operated from 1996 until June 2004. The data were downloaded in 2017 from https://archive.epa.gov/uvnet/web/html/access.html, accessed on 10 August 2021. The nine sites from the UV-Net network are Acadia National Park (ME), Albuquerque (NM), Boulder (CO), Canyonlands National Park (UT), Chicago (IL), Gaithersburg (MD), Research Triangle Park (NC), Riverside (CA), and Big Bend (TX). A total of 408,563 spectra were downloaded. As of 2020, EPA no longer makes these data available for download. However, the World Ultraviolet and Ozone Data Center (WODC) houses these data slightly reformatted for consistency with other global observations. The independent dataset of solar spectra used to validate the linear model was from the NIWA UV spectrometer systems, serial numbers UV3 (Mauna Loa, HI) and UV5 (Boulder, CO) in the USA, owned and operated by NOAA/ESRL/Global Monitoring Division, Boulder CO. They are maintained, calibrated, and operated by NOAA. The final data from both of these instruments are quality controlled and produced by NIWA-Lauder, New Zealand. The data are archived at the NDACC data repository, ftp://ftp.cpc.ncep.noaa.gov/ndacc/station/, accessed on 10 August 2021. The Solar-ISS spectrum was downloaded from http://bdap.ipsl.fr/voscat/SOLAR_ISS_V1.html in September 2020. Parsed in R using the library XML (version 3.99-03) routines htmlParse, getNodeSet, and readHTMLTable. The ozone absorption cross-section spectra were downloaded from http://www.atmos-meas-tech.net/6/3055/2013/amt-6-3055-2013.pdf on 31 August 2020, and read into R using read.table(skip = 21). For plots in this manuscript, we use the data from 273 °K, which provides data in units of cm^2^/molecule. To aid in reproducibility, the R-code used to create all figures is provided at https://github.com/SHADE-io/uvnet310, accessed on 10 August 2021, along with detailed instructions on obtaining the relevant datasets.

## References

[1] Diffey B.L. (2018). Time and Place as Modifiers of Personal UV Exposure. Int. J. Environ. Res. Public Health.

[2] US Department of Health and Human Services (2014). The Surgeon General’s Call to Action to Prevent Skin Cancer.

[3] Banerjee S., Hoch E.G., Kaplan P.D., Dumont E.L.P. A Comparative Study of Wearable Ultraviolet Radiometers. Proceedings of the 2017 IEEE Life Sciences Conference (LSC).

[4] Corrêa M.D.P., Godin-Beekmann S., Haeffelin M., Brogniez C., Verschaeve F., Saiag P., Pazmiño A., Mahé E. (2010). Comparison between UV Index Measurements Performed by Research-Grade and Consumer-Products Instruments. Photochem. Photobiol. Sci..

[5] Huang X., Chalmers A.N. (2021). Review of Wearable and Portable Sensors for Monitoring Personal Solar UV Exposure. Ann. Biomed. Eng..

[6] Zou W., Sastry M., Gooding J.J., Ramanathan R., Bansal V. (2020). Recent Advances and a Roadmap to Wearable UV Sensor Technologies. Adv. Mater. Technol..

[7] McKinlay A.F., Diffey B.L. (1987). A Reference Action Spectrum for Ultraviolet Induced Erythema in Human Skin. CIE J..

[8] Heckman C.J., Liang K., Riley M. (2019). Awareness, Understanding, Use, and Impact of the UV Index: A Systematic Review of over Two Decades of International Research. Prev. Med..

[9] WHO (2017). Global Solar UV Index.

[10] U.S. EPA, Exposure, and Atmospheric Sciences, EPA UV NET Ultraviolet Monitoring Program. https://archive.epa.gov/uvnet/web/html/access.html.

[11] Dumont E., Kaplan P. (2020). Methods, Systems, and Apparatuses for Accurate Measurement of Health Relevant UV Exposure from Sunlight. U.S. Patent.

[12] Huber M., Blumthaler M., Ambach W., Staehelin J. (1995). Total Atmospheric Ozone Determined from Spectral Measurements of Direct Solar UV Irradiance. Geophys. Res. Lett..

[13] U (2002). V. Global Solar, Index: A Practical Guide.

[14] (2015). U. S. EPA and OAR, UV Index.

[15] McKenzie R.L., Lucas R.M. (2018). Reassessing Impacts of Extended Daily Exposure to Low Level Solar UV Radiation. Sci. Rep..

[16] Allen M., McKenzie R. (2005). Enhanced UV Exposure on a Ski-Field Compared with Exposures at Sea Level. Photochem. Photobiol. Sci..

[17] Meftah M., Damé L., Bolsée D., Hauchecorne A., Pereira N., Sluse D., Cessateur G., Irbah A., Bureau J., Weber M. (2018). SOLAR-ISS: A New Reference Spectrum Based on SOLAR/SOLSPEC Observations. Astron. Astrophys. Suppl. Ser..

[18] Diffey B.L. (1975). Letter: Variation of Erythema with Monochromator Bandwidth. Arch. Dermatol..

[19] Berger D.S. (1976). The Sunburning Ultraviolet Meter: Design and Performance. Photochem. Photobiol..

[20] Hall E.S. (2009). Ground-Based Measurement of Solar Ultraviolet Radiation. Access Sci..

[21] Godar D.E., Wengraitis S.P., Shreffler J., Sliney D.H. (2001). UV Doses of Americans. Photochem. Photobiol..

[22] Gueymard C.A., Myers D., Emery K. (2002). Proposed Reference Irradiance Spectra for Solar Energy Systems Testing. Sol. Energy.

